# Diagnostic and Operative Challenges in a Type I First Branchial Cleft Cyst: A Case Report

**DOI:** 10.7759/cureus.32576

**Published:** 2022-12-15

**Authors:** Muhammad Sallehin Zulkaflay, Linda Lim Pei Fang, Yong Doh Jeing, Abd Razak Ahmad, Hardip Gendeh

**Affiliations:** 1 Department of Otorhinolaryngology-Head and Neck Surgery, Faculty of Medicine, Universiti Kebangsaan Malaysia, Kuala Lumpur, MYS; 2 Department of Otorhinolaryngology-Head and Neck Surgery, Hospital Melaka, Melaka, MYS

**Keywords:** congenital cyst, parotid tumor, neck mass, pre-auricular cyst, first branchial cleft

## Abstract

First branchial cleft cyst (FBCC) is a rare entity of congenital anomalies in the head and neck area. Dealing with FBCC is a clinical challenge as the condition is frequently forgotten in the differential diagnosis of lateral neck swelling. We report a rare case of unilateral type I FBCC in an 11-year-old boy who presented with a painless and slow-growing preauricular mass masquerading as a benign cystic lesion of the parotid. The lesion was completely removed via surgical excision. Histopathology report confirmed the findings of squamous epithelium‑lined cyst wall, which was a characteristic of a branchial cleft cyst. The combination of good clinical acumen, with the help of radiological correlation, along with a strong degree of suspicion for the condition, facilitates the diagnosis of this condition and hence proper management.

## Introduction

Among the congenital neck masses in the pediatric population, branchial cleft anomalies are the second most common following thyroglossal duct cysts. Within the diagnosis of branchial anomalies, the second branchial cleft is the most affected, with a reported incidence of up to 95% of cases [1]. The first branchial cleft anomalies, however, are extremely rare and comprise less than 1% of all branchial anomalies [2]. They have an estimated incidence of 1 in 100,000 [3].

Work has classified first branchial cleft cyst (FBCC) into two types [4]. Type I, which is ectodermal in origin, is regarded to be a duplication of the membranous external auditory canal (EAC), containing only squamous epithelium. It usually forms a cyst adjacent to the EAC. Type II originates from both the mesoderm and ectoderm, containing squamous epithelium, cartilage, and skin appendage. They may present as cysts, sinuses, or fistula in the region of the angle of the mandible.

The diagnosis of FBCC, prior to surgery, is mostly based on clinical signs and symptoms. Due to their rarity and unfamiliar clinical features, even with the aid of radiological images, they can be easily misdiagnosed, leading to improper treatment. This case report highlights how an FBCC presenting as a benign parotid tumor. Besides, the challenges we faced in its diagnosis and surgery are also described.

## Case presentation

**Figure 1:**
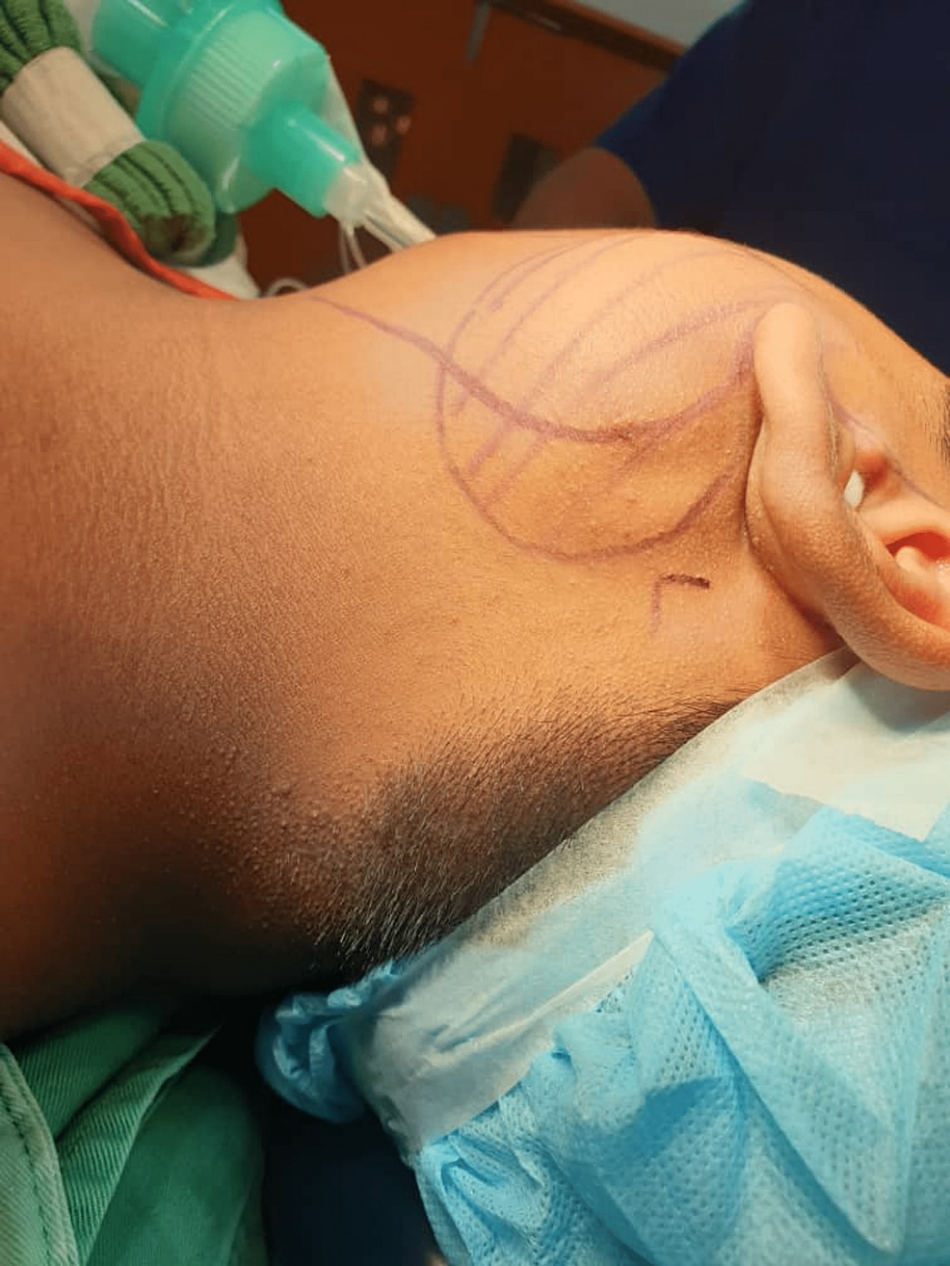
A well-defined mass in the intra-auricular region of the left neck measuring 5x5cm.

An 11-year-old boy with no known medical illness was referred to our otorhinolaryngology clinic. He presented with a painless swelling in the left neck since he was two years old. It was slowly increasing in size over the last two years, which prompted him to seek medical attention. There was no history of recurrent ear discharge. Upon physical examination, there was a well-defined mass in the parotid region of the left neck measuring 5x5cm (Figure 1). It was cystic in nature and non-tender with no skin changes. Other examination findings were unremarkable.

**Figure 2:**
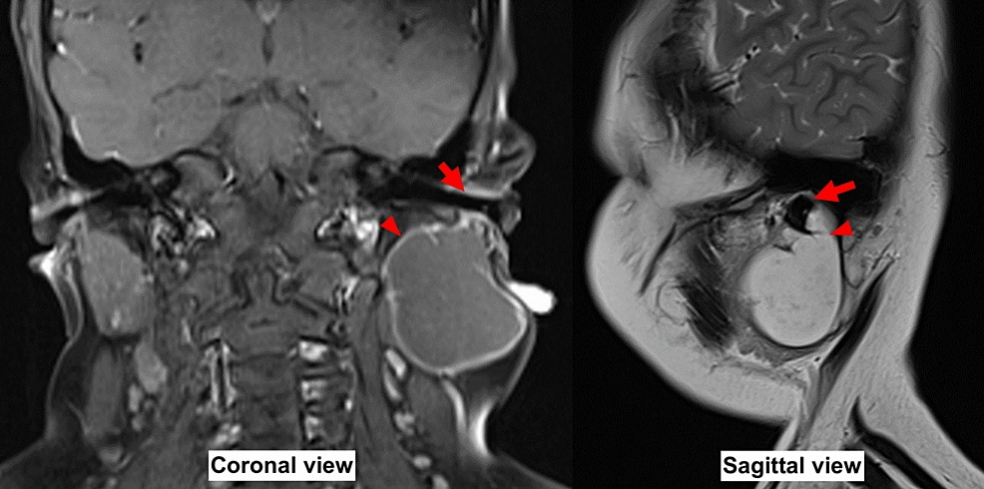
MRI of the neck revealing a well-defined lobulated intra-glandular cystic lesion (arrowhead) within the left parotid gland measuring 4x4cm near the external auditory canal (arrow). First image in T1 coronal view showing a hypointense lesion. Second image in T2 sagittal view showing a hyperintense lesion.

An ultrasound of the neck was suggestive of a cystic lesion of the left parotid tissue. Magnetic resonance imaging (MRI) was performed for further evaluation, which demonstrated a well-defined lobulated intra-glandular cystic lesion within the left parotid gland measuring 4x4cm (Figure 2). A fine needle aspiration of the lesion revealed an acellular material containing degenerated squamous cells.

**Figure 3:**
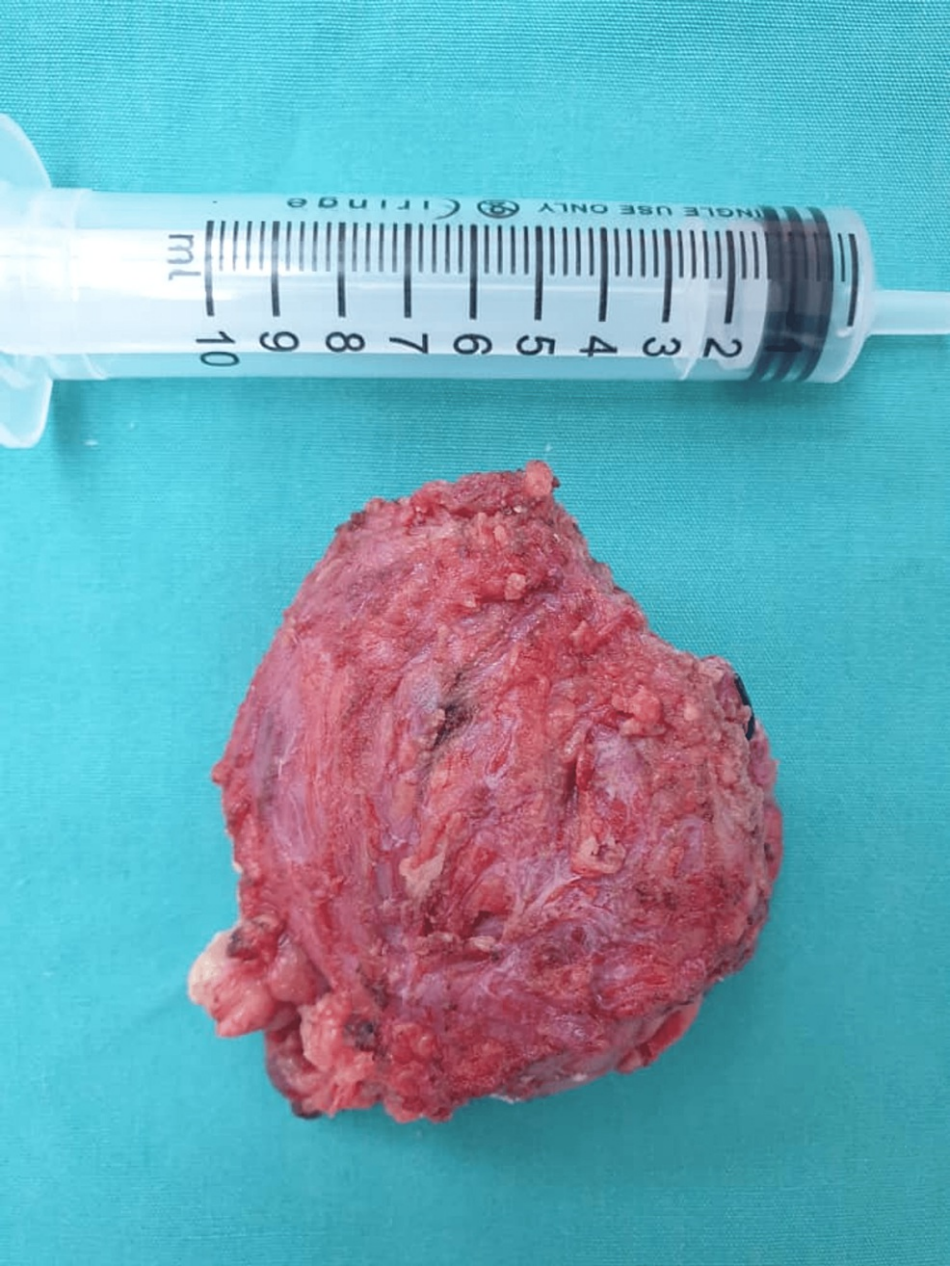
The cystic lesion measured 50mm in diameter with residual parotid tissue.

**Figure 4:**
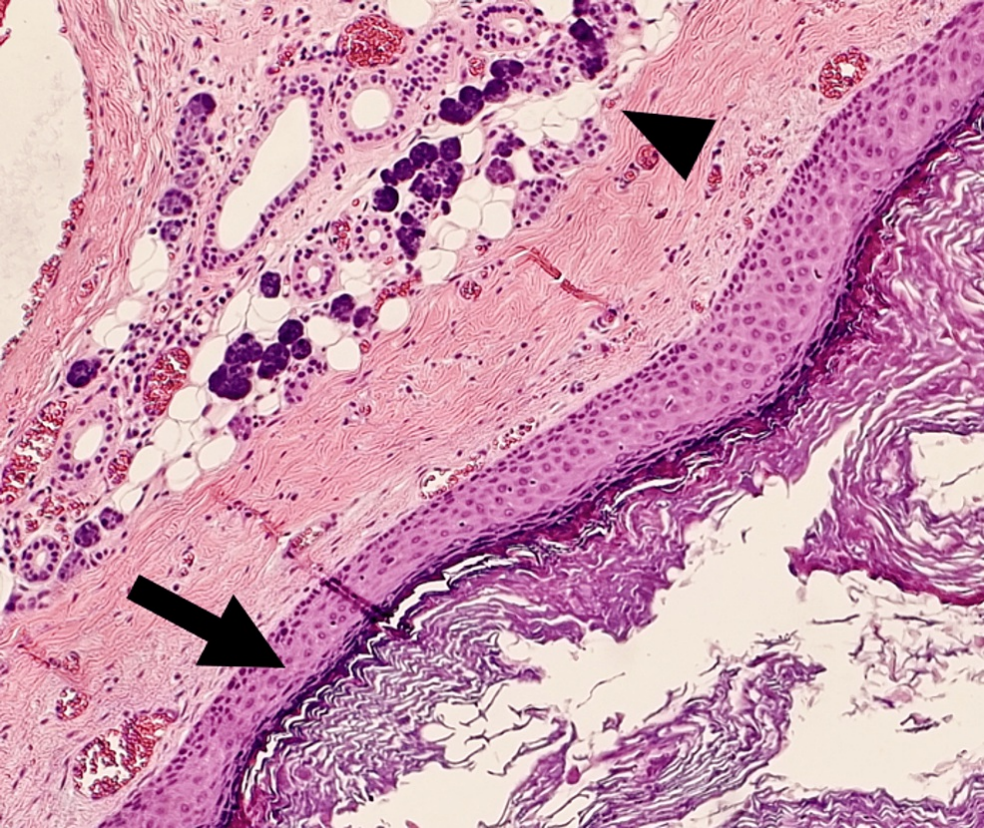
Histopathology showing the cyst lined by keratinizing stratified squamous epithelium and filled with keratin squamous cells (arrow) with the adjacent parotid tissue (arrowhead).

With a provisional diagnosis of a benign cystic lesion of the parotid, he underwent a superficial parotidectomy. Intra-operatively, the cystic lesion was located within the superficial lobe of the left parotid gland superficial and lateral to the facial nerve. Difficulties were encountered as we noticed that the cyst can be traced superiorly to and adhered firmly to the cartilaginous part of the EAC. Believed to be a duplicate of EAC, this raised our suspicion of an FBCC. The lesion was carefully explored, and the cyst was removed together with a small section of the EAC cartilage. Remaining of the EAC cartilage was primarily closed, all the facial nerve branches were identified and preserved, and the lifted superficial lobe was replaced. The histopathology reported a cystic lesion measuring 50mm in diameter on sectioning with residual glandular tissue (Figure 3). The cyst was lined by keratinizing stratified squamous epithelium and filled with keratin squamous cells, which confirmed the diagnosis of a type I FBCC (Figure 4). Post-operatively, the incision on the neck and EAC defect were healing well, and there was mild facial nerve paresis. Under our surveillance follow-up, the cosmesis was satisfactory, and he completely recovered from the facial paresis after three months. There was no otologic complication and no sign of recurrence one year post-surgery.

## Discussion

Neck masses in pediatric medicine are commonly encountered and can position the clinician involved in a diagnostic dilemma. The common location for neck swelling indicating an FBCC includes the cervical (41%), parotid (35%), and preauricular regions (24%) [5]. It is caused by failure of obliteration of the cervical sinus of His during the embryonic development. During development, the obliteration of the branchial cleft occurs concurrently with the development of the facial nerve and the parotid gland, which originate from the second branchial arch [6]. This explains the close relationship between the first branchial cleft anomalies and these structures, as described in the present case.

Clinically, it is challenging to diagnose FBCC. Their rarity and similar presentation with other conditions such as chronic otitis externa, abscess, parotid tumor, or tail of parotid cyst often lead to misdiagnosis, resulting in inappropriate treatment. In our case, it mimicked a parotid tumor. While parotid swelling will be present in both cases, FBCC has a various spectrum of manifestations (parotid, cervical, and auricular), which may differentiate them from the parotid tumor [7]. The mass may be accompanied by the presence of sinus or fistula in the neck. History of repeated episodes of infection with multiple incision and drainage is pathognomic of type II FBCC [8]. For otologic manifestation, the most frequent presentation is purulent discharge from the EAC. Presence of any fistula in the EAC must be excluded. The EAC may also be partially or completely obstructed by the cystic swelling pushing the canal walls. These, however, can be completely absent in an isolated cyst. This is why in our case, the lack of other manifestations added to the difficulty in the diagnosis.

Diagnostic modalities may help provide substantial information in a patient with parotid mass, but their accuracy for diagnosis is sometimes questionable, especially in differentiating rare disease. Ultrasonography can be of first assistance, which could ascertain the cystic nature of the lesion. An ultrasound Doppler would be useful if a vascular lesion is suspected to assess flow. MRI will be able to help in providing valuable information about the lesion and anatomical relation, suggesting the possible condition, and guiding the surgical approach owing to its high resolution in soft tissue differentiation. There is no specific radiological feature for the diagnosis of FBCC as opposed to other benign cysts [9]. However, being adjacent to the EAC or the presence of fistula or sinus should prompt the thought of the diagnosis. Apart from imaging, fine-needle aspiration cytology can assist in the preoperative diagnosis, helping in exclusion of the presence of malignancy or inflammatory adenopathy.

The treatment modality of choice for FBCC is surgical excision. Ideally, the access is by parotidectomy approach using modified Blair incision with adequate exposure of the facial nerve trunk [10]. In some cases, resection of a small amount of skin and EAC cartilage is needed for permanent cure [11]. Recurrence occurs if the lesion is not completely excised along its entire course. According to a study, facial nerve palsy is the most common complication with an incidence rate of 25% followed by a recurrence rate of 13%, infection rate of 8%, and EAC stenosis rate of 1% [12]. The need for meticulous exploration and identification of both the facial nerve and the whole lesion is surgically challenging, considering the complication in the two extremes. Recurrence may occur if we are too much concerned about avoidance of facial nerve damage. On the other hand, facial nerve palsy may occur if the concern is more for making sure that no residual lesion is left. The use of facial nerve monitoring intra-operatively may help to alleviate risk of injury to the facial nerve.

## Conclusions

FBCC is a rare entity. It demands a great attention to the presenting history and physical examination together with thorough pre-operative radiological work-up in considering the condition. The presence of a parotid cyst in a pediatric age group with radiological correlation of being adjacent to the EAC should raise the level of suspicion. The complexity and close relationship of FBCC with facial nerve and EAC must not be underestimated by the treating surgeons.
